# Arthroscopic Safe Trans‐subscapularis Portal for Bony Bankart Management

**DOI:** 10.1002/atn2.70331

**Published:** 2026-10-22

**Authors:** Ziyad El Qirem, Sameer Adel, Sanad Alqawasmi, Ahmad AlZoubi

**Affiliations:** ^1^ Department of Orthopedic Surgery The Specialty Hospital Amman Jordan

## Abstract

Bony Bankart lesions are a relatively common pathology encountered in patients with anterior shoulder dislocation. Surgical management is influenced by multiple factors, including the size, chronicity of the bony fragment, fragment quality, and patient‐specific considerations such as age, activity level, and risk of recurrence. Several arthroscopic fixation techniques have been described for bony Bankart lesions. Regardless of the fixation method, portal strategy plays a critical role in achieving safe and effective fragment reduction and fixation. In this technical note, we describe an arthroscopic accessory anterior portal for bony Bankart management. This portal provides optimal access to the anteroinferior glenoid, facilitates the instrument trajectory for fixation, and maintains a safe distance from the axillary nerve. The described technique aims to enhance surgical efficiency while improving safety during arthroscopic bony Bankart management.

## Graphical abstract

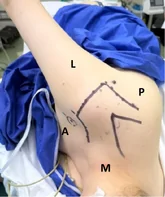

**VIDEO 1:** Arthroscopic safe trans‐subscapularis portal for bony Bankart management. The video is a case example of arthroscopic management of bony Bankart lesion of the right shoulder with the patient in lateral position right side up using the safe trans‐subscapularis portal as the working portal, anterosuperior portal as the main viewing portal after opening the rotator interval, and posterior portal as the initial viewing portal for diagnostic arthroscopy and opening the rotator interval. The following steps are included: (1) diagnostic arthroscopy, (2) opening the rotator interval, (3) clearing the borders of the subscapularis tendon, (4) releasing and rasping the bony Bankart fragment, (5) reduction sutures, (6) insertion of the medial glenoid surface anchor, (7) placement of suture around the bony Bankart fragment using the safe trans‐subscapularis tendon, (8) suture retrieval out of the subscapularis tendon, and (9) suture fixation. Video content can be viewed at https://doi.org/10.1002/atn2.70331.

Bony Bankart lesions are characterized by avulsion of the anteroinferior labroligamentous complex in association with a fracture of the anterior glenoid rim. These injuries most commonly occur after traumatic anterior shoulder dislocation and represent a significant contributor to recurrent glenohumeral instability.^1^ Failure to recognize and adequately address the osseous component may result in persistent instability, recurrent dislocation, and progressive glenoid bone loss.

Although open reduction and internal fixation have historically been used for large or displaced fragments, open techniques require extensive surgical exposure and soft‐tissue dissection. This approach has been associated with increased postoperative pain, stiffness, and potential subscapularis dysfunction.^2^

With advances in instrumentation and surgical expertise, arthroscopic management has become increasingly favored. Arthroscopic reduction and fixation allow for anatomic restoration of the glenoid rim while minimizing soft‐tissue disruption. Furthermore, arthroscopy enables comprehensive intra‐articular assessment and treatment of associated pathologies.^3^, ^4^, ^5^ Despite these advantages, arthroscopic fixation of bony Bankart lesions remains technically demanding. Achieving adequate visualization, obtaining safe medial access to the fragment, and placing fixation devices without compromising the surrounding neurovascular structures present significant challenges.

In this technical note, we describe a reproducible arthroscopic technique for the management of bony Bankart lesions using a safely established anterior accessory portal under direct visualization. This approach facilitates improved medial access and fragment control, enhances exposure of the fracture bed, and allows for secure and stable fixation while minimizing the risk of iatrogenic injury.

## SURGICAL TECHNIQUE

### Preoperative Evaluation

Patients with a bony Bankart lesion usually present with anterior shoulder pain and a feeling of instability during range of motion, with a preceding history of anteroinferior shoulder dislocation. The apprehension and relocation tests are usually positive. The diagnosis can be made with the help of radiograph, computed tomography scan, and magnetic resonance imaging because these modalities better delineate the pathology and evaluate any associated conditions, such as rotator cuff tears.

### Setup

After the induction of general anesthesia and administration of an interscalene block, the patient is placed on the operating table in the lateral decubitus position with the operative side up. After preparation and draping of the shoulder, the arthroscopic portals are marked on the operative shoulder (Figure 1).

**FIGURE 1:**
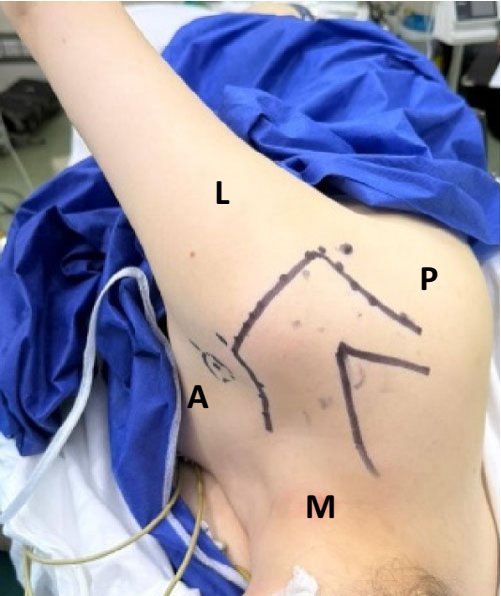
An orientation of the patient position as A, P, L, and M. The patient is in lateral position right side up with one side support at the sacrum and another one is just distal to the inferior pole of the scapula, an axillary role is placed under the contralateral shoulder, and pillow between the legs and hard pillow under the head and traction system Trimano (Arthrex, Naples, FL) are used on the anterior side of the patient. (A, anterior; L, lateral; M, medial; P, posterior.)

### Portals

This technique is performed using a 30° arthroscope. Three arthroscopic portals are used: a standard posterior portal (located 2 cm inferior and 1 cm medial to the posterolateral corner of the acromion) as the initial viewing portal, the safe trans‐subscapularis portal, and an anterosuperior portal (Figure 2). The operative technique is shown in Video 1.

**FIGURE 2:**
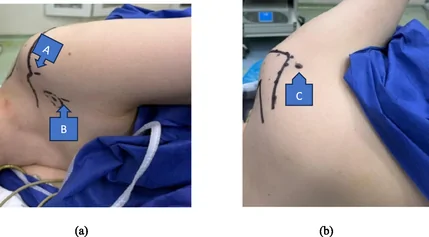
The patient is in a lateral position and right shoulder is shown. (a) The anterior portals are marked as (A) anterosuperior portal (just medial to the anterolateral edge of the acromion and under direct visualization just anterior to the long head of the biceps tendon), (B) safe trans‐SSC portal (just lateral to the coracoid process under direct visualization at the upper border of the SSC tendon). (b) The posterior portal is marked by (C) (located 2 cm inferior and 1 cm medial to the posterolateral corner of the acromion). (SSC, subscapularis.)

### Operation

#### Diagnostic Arthroscopy

A standard diagnostic shoulder arthroscopy is performed. Starting from the posterior viewing portal, after confirming the diagnosis of a bony Bankart lesion, the safe trans‐subscapularis portal is established just lateral to the coracoid process under direct visualization at the upper border of the subscapularis tendon as the working portal. Then, after complete opening of the rotator interval, an anterosuperior portal is created just medial to the anterolateral edge of the acromion and under direct visualization just anterior to the long head of the biceps tendon, and the arthroscope is switched to this portal. Care should be taken to maintain an adequate skin bridge between the anterior portals to avoid instrument crowding. A 7‐mm rigid Twist‐In cannula (Arthrex, Naples, FL) is then inserted into the posterior portal for suture management.

#### Opening the Rotator Interval

The procedure begins with complete opening of the rotator interval using a radiofrequency ablator Apollo (Arthrex), with clearance of the anterior, superior, and posterior borders of the subscapularis tendon using the posterior viewing portal and the safe trans‐subscapularis working portal (Figures 3 and 4).

**FIGURE 3:**
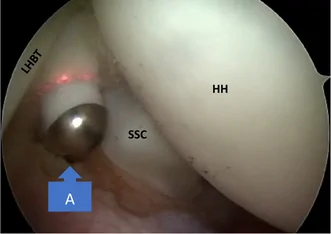
Viewing the glenohumeral joint from the posterior portal while the patient is in lateral position right side up. (A) This explains the main idea and use of the safe trans‐SSC portal during complete opening of the RI using the radiofrequency ablator Apollo (Arthrex, Naples, FL) through the safe trans‐SSC portal. (HH, humeral head; LHBT, long head biceps tendon; RI, rotator interval; SSC, subscapularis.)

**FIGURE 4:**
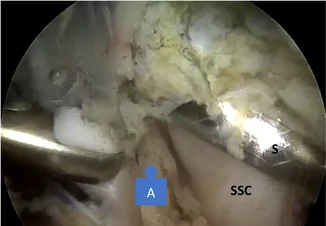
The arthroscope is changed to the anterosuperior portal, and a 7‐mm rigid Twist‐In cannula (Arthrex, Naples, FL) is then inserted into the posterior portal for suture management and now viewing the glenohumeral joint from the anterosuperior portal while the patient is in lateral position right side up. SSC tendon and S are used for traction. (A) Clearing the borders of the SSC tendon using the radiofrequency ablator Apollo (Arthrex) through the safe trans‐SSC portal. (S, switching stick; SSC, subscapularis.)

#### Bony Bankart Preparation

After opening the interval, the arthroscope is switched to the anterosuperior portal; while working from the safe trans‐subscapularis portal, the bony Bankart fragment is released from the glenoid using an arthroscopic periosteal elevator and radiofrequency ablator Apollo (Arthrex) down to the 6‐o'clock position inferiorly and medially to the fibers of the subscapularis muscle. Rasping of the glenoid surface is then performed (Figure 5).

**FIGURE 5:**
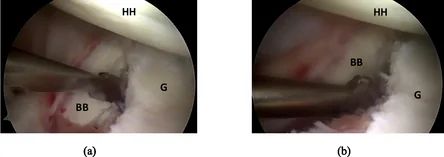
Viewing the glenohumeral joint from the anterosuperior portal while the patient is in lateral position right side up. (a) Release of the BB using the arthroscopic periosteal elevator. (b) Rasping of the G anteroinferior surface using arthroscopic rasp through the safe trans‐SSC portal. (BB, bony Bankart fragment; G, glenoid; HH, humeral head; SSC, subscapularis.)

#### Suture Management

Two sutures are placed in a loop fashion through the anterior labrum using a 25° SutureLasso (Arthrex) through the safe trans‐subscapularis portal at the inferior and superior ends of the bony Bankart fragment and are used for reduction. Both sutures are retrieved through the posterior portal to assess the reduction (Figure 6). After retrieving the reduction sutures from the safe trans‐subscapularis portal, a double‐loaded 2.9‐mm PEEK (polyether ether ketone) knotted anchor SutureTak (Arthrex) is then placed on the medial surface of the glenoid, and the sutures are retrieved through the posterior portal (Figure 7).

**FIGURE 6:**
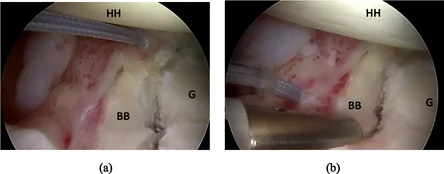
Viewing the glenohumeral joint from the anterosuperior portal while the patient is in lateral position right side up. (a) Loop suture is taken in the inferior part of BB as reduction suture using 25° SutureLasso (Arthrex, Naples, FL) from the safe trans‐SSC portal. (b) Another reduction suture in the superior part of the BB is taken in the same technique as (a), and then the sutures are shuttled through the posterior portal. (BB, bony Bankart fragment; G, glenoid; HH, humeral head; SSC, subscapularis.)

**FIGURE 7:**
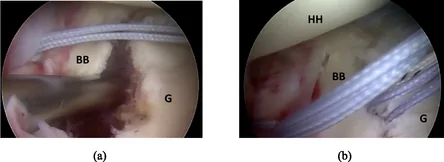
Viewing the glenohumeral joint from the anterosuperior portal while the patient is in lateral position right side up. (a) A 2.9‐mm knotted anchor PushLock (Arthrex, Naples, FL) guide is placed through the safe trans‐SSC portal on the medial surface of the G. (b) Anchor sutures are retrieved through the posterior portal. (BB, bony Bankart fragment; G, glenoid; HH, humeral head; SSC, subscapularis.)

A 45° SutureLasso (Arthrex) is used. The SutureLasso (Arthrex) is introduced from the safe trans‐subscapularis portal through the rotator interval, and it passes through the upper tendinous part of subscapularis tendon under direct visualization of the anterior and posterior borders of the tendon and lateral to the coracoid, then the SutureLasso (Arthrex) is passed medial to the bony Bankart fragment at its inferior end, and the shuttling wire is retrieved through the posterior portal. Shuttling of the 1 blue and 1 tiger sutures is performed. Using the same suture management technique, another bite is taken at the superior end of the bony Bankart fragment using the other blue and tiger sutures (Figure 8); this explains the main idea and use of the safe trans‐subscapularis portal. The sutures are then retrieved out the subscapularis tendon from inside the glenohumeral joint to the safe trans‐subscapularis portal (Figure 9).

**FIGURE 8:**
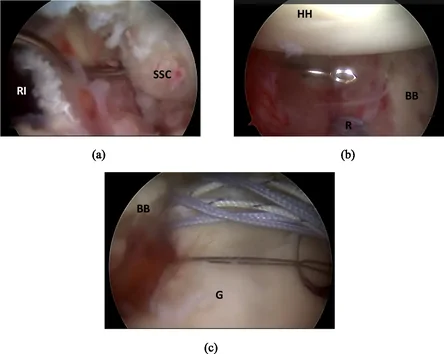
Explains the use of the safe trans‐SSC portal, viewing the glenohumeral joint from the anterosuperior portal while the patient is in lateral position right side up. (a) A 45° SutureLasso (Arthrex, Naples, FL) is introduced through the safe trans‐SSC portal to the RI and then passed through the anterior surface of the SSC tendon. (b) The SutureLasso (Arthrex) is passed through the posterior surface of the SSC tendon inside the glenohumeral joint. (c) The SutureLasso (Arthrex) is passed inferomedially to the BB; then, the shuttling wire is retrieved from the fracture site through the posterior portal. (BB, bony Bankart fragment; G, glenoid; HH, humeral head; R, superior reduction suture; RI, rotator interval; SSC, subscapularis.)

**FIGURE 9:**
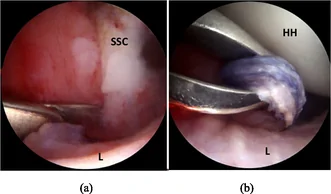
Viewing the glenohumeral joint from the anterosuperior portal while the patient is in lateral position right side up. (a,b) The blue repair sutures retrieved out of the SSC tendon from inside the glenohumeral joint through the safe trans‐SSC portal. (HH, humeral head; L, labrum; SSC, subscapularis.)

Reduction is then achieved using the reduction sutures. Through the safe trans‐subscapularis portal, the inferior reduction sutures and the blue sutures from the anchor on the medial glenoid surface are fixed to the glenoid rim at the 5‐o'clock position using a 3.5‐mm knotless PEEK anchor PushLock (Arthrex). The tiger sutures are fixed to the glenoid rim at the 3‐o'clock position using 2.0 knotless PEEK anchor PushLock (Arthrex) (Figure 10). The superior reduction suture is then removed. At the end, there are 4 sutures going from the medial surface of the glenoid and around the bony Bankart fragment then fixed to the glenoid rim (Figure 11).

**FIGURE 10:**
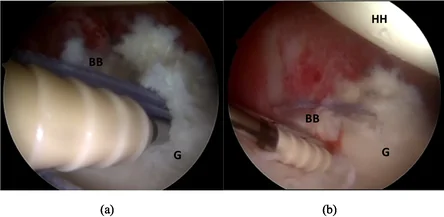
Viewing the glenohumeral joint from the anterosuperior portal while the patient is in lateral position right side up. (a,b) The repair suture fixation to the G rim through the safe trans‐SSC portal. (a) The inferior reduction sutures and the blue sutures from the anchor on the medial G surface are fixed to the G rim at the 5‐o'clock position using a 3.5‐mm knotless PEEK anchor PushLock (Arthrex, Naples, FL). (b) The tiger sutures from the anchor on the medial surface of the G are fixed to the G rim at the 3‐o'clock position using 2.0 knotless PEEK anchor PushLock (Arthrex). (BB, bony Bankart fragment; G, glenoid; HH, humeral head; PEEK, polyether ether ketone; SSC, subscapularis.)

**FIGURE 11:**
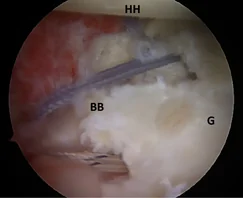
Viewing the glenohumeral joint from the anterosuperior portal while the patient is in lateral position right side up. The end result after the repair with 4 sutures around the bony Bankart reducing and fixing it to the G rim and inferior reduction suture augmenting the construct. (BB, bony Bankart fragment; G, glenoid; HH, humeral head.)

## DISCUSSION

Bony Bankart lesions represent a spectrum of anterior glenoid rim injuries associated with traumatic anterior shoulder instability. The optimal surgical management remains controversial. Although both open and arthroscopic techniques have shown favorable outcomes, arthroscopic management has gained widespread acceptance.^3^, ^4^

Restoration of the native glenoid anatomy through anatomic reduction and stable fixation helps re‐establish the articular arc and capsulolabral tension, which are critical for preventing recurrent instability. In addition, arthroscopy facilitates the management of associated lesions, including capsulolabral tears, cartilage injuries, and Hill‐Sachs defects, in a single setting.^4^, ^5^

Despite these advantages, arthroscopic management presents technical challenges. One of the most significant difficulties is achieving adequate access to the medial aspect of the fragment, especially when the fragment is large or displaced medially. The arthroscopic suture bridge technique for patients with bony Bankart lesions is associated with excellent shoulder function, low pain levels, and high return‐to‐sport rates at long‐term follow‐up.^6^, ^7^, ^8^ But limited visualization and working angles may increase the risk of fragment comminution or suboptimal reduction. Portal selection is therefore crucial. Various anterior, anteroinferior, and trans‐subscapularis portals have been described to improve trajectory and access; however, each carries potential risks, including iatrogenic injury to the subscapularis tendon or neurovascular structures. Here, we describe a technique that addresses these difficulties by fixing the fragment using sutures around it, thereby avoiding damage to the fragment or the subscapularis tendon. Pearls and pitfalls are listed in Table 1.

**TABLE 1:** Pearls and Pitfalls

**Step**	**Pearls**	**Pitfalls**
Opening the rotator interval and without using a cannula	• Allows safe, controlled and under direct visualization of the SutureLasso while it passes through the subscapularis tendon • Not using a cannula gives a more flexibility in the lasso trajectory	• Extravasation of fluid to the anterior compartment • Soft‐tissue bridge can develop
Passing the SutureLasso through the subscapularis tendon	Gives more medial access to the bony fragment, and once the lasso is inside the joint, it will be safe and away from the axillary nerve	More demanding suture management

A few limitations of this technique include a possible high learning curve for the surgeon. The advantages and disadvantages of this technique are listed in Table 2.

**TABLE 2:** Advantages and Disadvantages

**Advantages**	**Disadvantages**
Safe portal that reduces the risk of neurovascular injury	Technically demanding and needs skills in using the SutureLasso and suture management
Allows more medial instrumentation	The need for 2 anterior portals
Avoid damage to the subscapularis tendon	

## DISCLOSURES

The authors (Z.E.Q., S.A., S.A., A.A.) declare that they have no known competing financial interests or personal relationships that could have appeared to influence the work reported in this paper.

## References

[1] Weisberg Z , Cole W , Rumps MV , Vopat B , Mulcahey MK . Bony Bankart lesion: Diagnosis, management, and outcomes. JBJS Rev. 2024;12:e23. 10.2106/JBJS.RVW.23.00200 38709911

[2] Wang L , Wang H , Li X , et al. A meta‐analysis of arthroscopic versus open repair for treatment of Bankart lesions in the shoulder. Med Sci Monit. 2015;21:3028‐3035. 10.12659/MSM.894346 PMC4603609 26446430

[3] Chen AZ , Greaves KM , deMeireles AJ , Fortney TA , Saltzman BM , Trofa DP . Clinical outcomes of arthroscopic bony Bankart repair for anterior instability of the shoulder: A systematic review. Am J Sports Med. 2023;51:2758‐2765. 10.1177/03635465221094832 35749344

[4] Kitayama S , Sugaya H , Takahashi N , et al. Clinical outcome and glenoid morphology after arthroscopic repair of chronic osseous Bankart lesions: A 5‐ to 8‐year follow‐up study. J Bone Joint Surg Am. 2015;97:1833‐1843. 10.2106/JBJS.N.01033 26582613

[5] Godin JA , Altintas B , Horan MP , et al. Midterm results of the bony Bankart bridge technique for the treatment of bony Bankart lesions. Am J Sports Med. 2019;47:158‐164. 10.1177/0363546518808495 30485124

[6] Hinz M , Kruckeberg BM , Davis CS , et al. Minimum 10‐year clinical and functional outcomes after arthroscopic bony Bankart bridge for the treatment of bony Bankart lesions. J Shoulder Elbow Surg. 2025;34(suppl 6):S57‐S63. 10.1016/j.jse.2025.02.002 39984032

[7] Qu D , Fu H , Shen Y , et al. Modified double‐pulley fixation provides better reduction of bone fragments and union compared to single‐point fixation in bony Bankart lesions. Knee Surg Sports Traumatol Arthrosc. 2024;32:2141‐2151. 10.1002/ksa.12218 38721628

[8] Osun MF , Oklaz EB , Ahmadov A , et al. Early surgical stabilization is a positive predictor whereas dominant extremity involvement and off‐track lesions are negative predictors in arthroscopic repair of bony Bankart lesions with glenoid bone loss <25%. Arthroscopy. 2025;41:4472‐4482. 10.1016/j.arthro.2025.05.035 40482980

